# An Integrative Platform for Three-dimensional Quantitative Analysis of Spatially Heterogeneous Metastasis Landscapes

**DOI:** 10.1038/srep24201

**Published:** 2016-04-12

**Authors:** Ian H. Guldner, Lin Yang, Kyle R. Cowdrick, Qingfei Wang, Wendy V. Alvarez Barrios, Victoria R. Zellmer, Yizhe Zhang, Misha Host, Fang Liu, Danny Z. Chen, Siyuan Zhang

**Affiliations:** 1 Department of Biological Sciences, College of Science, University of Notre Dame, Notre Dame, IN 46556, USA; 2 Mike and Josie Harper Cancer Research Institute, University of Notre Dame, 1234 N. Notre Dame Avenue, South Bend, IN 46617, USA; 3 Department of Computer Science and Engineering, College of Engineering, University of Notre Dame, Notre Dame, IN 46556, USA; 4 Department of Applied and Computational Mathematics and Statistics, College of Science, University of Notre Dame, Notre Dame, IN 46556, USA

## Abstract

Metastatic microenvironments are spatially and compositionally heterogeneous. This seemingly stochastic heterogeneity provides researchers great challenges in elucidating factors that determine metastatic outgrowth. Herein, we develop and implement an integrative platform that will enable researchers to obtain novel insights from intricate metastatic landscapes. Our two-segment platform begins with whole tissue clearing, staining, and imaging to globally delineate metastatic landscape heterogeneity with spatial and molecular resolution. The second segment of our platform applies our custom-developed SMART 3D (**S**patial filtering-based background removal and **M**ulti-ch**A**nnel forest classifiers-based 3D **R**econs**T**ruction), a multi-faceted image analysis pipeline, permitting quantitative interrogation of functional implications of heterogeneous metastatic landscape constituents, from subcellular features to multicellular structures, within our large three-dimensional (3D) image datasets. Coupling whole tissue imaging of brain metastasis animal models with SMART 3D, we demonstrate the capability of our integrative pipeline to reveal and quantify volumetric and spatial aspects of brain metastasis landscapes, including diverse tumor morphology, heterogeneous proliferative indices, metastasis-associated astrogliosis, and vasculature spatial distribution. Collectively, our study demonstrates the utility of our novel integrative platform to reveal and quantify the global spatial and volumetric characteristics of the 3D metastatic landscape with unparalleled accuracy, opening new opportunities for unbiased investigation of novel biological phenomena *in situ*.

Tumor metastasis is orchestrated by the interplay between genetically heterogeneous cancer cells and a spatially and compositionally heterogeneous tumor microenvironment (TME, also referred to as the metastatic niche)^1^,^2^,^3^,^4^,^5^. Metastatic cells and the metastatic TME – together, termed the metastatic landscape – are composed of several different cell types that display an ever-evolving heterogeneity throughout metastatic progression^6^,^7^,^8^. It has been envisioned that spatially compartmentalized metastatic niches differentially regulate metastatic progression^6^,^7^,^8^. For example, the role of astrogliosis during brain metastasis formation has been debated for decades^9^,^10^. Limited by two-dimensional (2D) *in vitro* culture and histology methods, previous studies were unable to fully describe the spatial heterogeneity of astrogliosis or deduce the functional implications of astrogliosis during brain metastasis progression *in situ* within large, intact tissue samples. Similarly, angiogenesis, a hallmark of cancer, is crucial to maintain brain tumor outgrowth, such as in gliomas^1^,^11^. Yet, the requirement and characterization of angiogenesis during brain metastasis progression remain largely controversial^10^. Because only a small fraction of the total vasculature can be captured in a single standard histological slice, even the most concrete brain metastasis vascularisation data draw speculative conclusions. Despite the significance of examining spatial aspects of heterogeneous metastases in their metastatic niche, technical barriers have impeded efforts to dissect the contribution of diverse spatial components of the metastatic landscape *in situ* on a global three-dimensional (3D) scale with molecular-level resolution^12^.

The recent boom of whole tissue clearing techniques presents us with an unprecedented opportunity to dissect metastatic heterogeneity *in situ*^13^. Tissue clearing permits a holistic, 3D view of tissue, which is particularly useful to image two metastatic landscape components – astrocytes and vasculature – that cannot be captured in a single plane and have diverse or highly speculated roles in metastatic progression^9^,^10^,^14^,^15^,^16^. Furthermore, the 3D perspective provided by tissue clearing allows spatial analysis, which can provide novel insights into biological phenomena. While tissue clearing provides an unmatched opportunity to explore the metastatic landscape, the massive volumetric datasets derived from whole tissue imaging impose new challenges on the image analysis of multiple genetic events with statistically significant biological implications. In this study, we developed and applied an integrative platform including a 3D whole brain imaging approach, consisting of whole tissue clearing, staining, and imaging, followed by customized computer-assisted quantifications. We developed SMART 3D (**S**patial filtering-based background removal and **M**ulti-ch**A**nnel forest classifiers-based 3D **R**econs**T**ruction), a multi-faceted image analysis pipeline, to observe and quantify phenotypic metastatic landscape heterogeneity *in situ* with spatial and molecular resolution. Our implementation of our integrative platform to globally analyze the heterogeneous metastasis landscape of brain metastases demonstrates the feasibility of quantitative, multiplexed 3D analysis *in situ* from the molecular level to the whole organ scale. Furthermore, our study asserts the promise of such analysis in revealing unique spatial patterns of metastasis that will lead to novel functional and molecular insights into the dynamic nature of metastasis.

## Results

### Global imaging of multiple metastatic landscape features with molecular resolution

**Figure 1:**
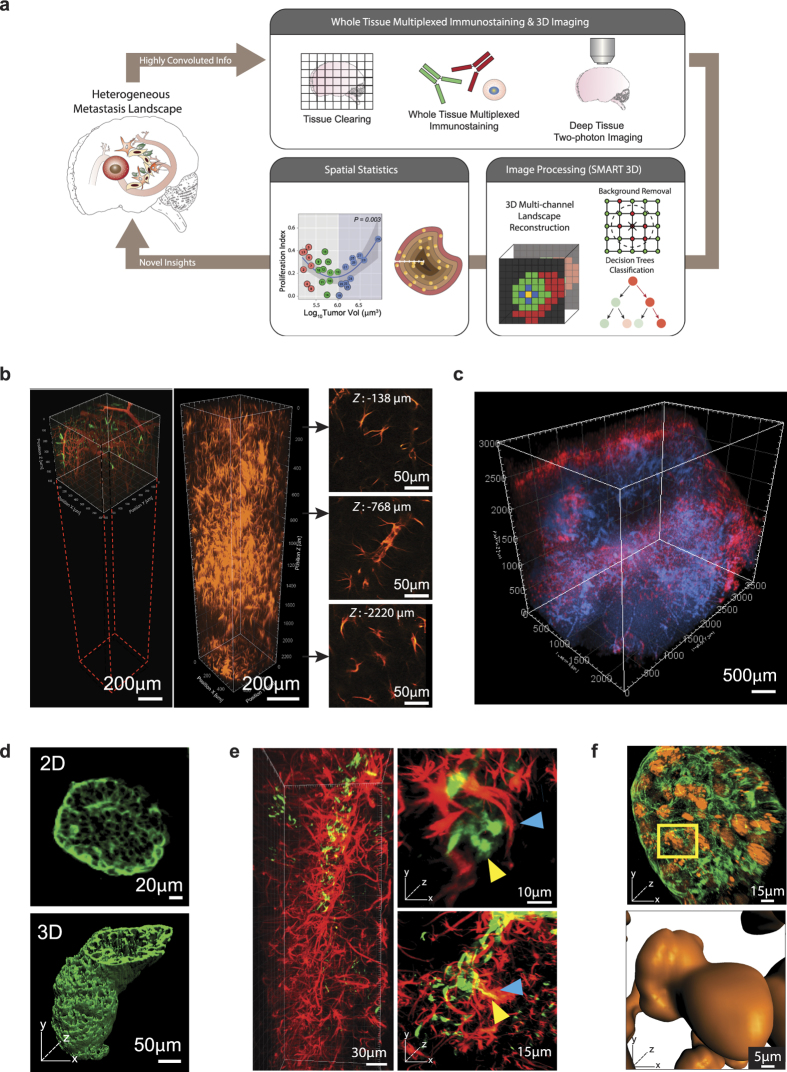
3D Whole Tissue Imaging of the Brain Metastasis Landscape with Molecular Resolution. (**a**) Schematic of our integrative platform consisting of whole tissue clearing, staining, and imaging and SMART 3D (image processing and quantification). (**b**) Comparison of multiphoton-imaging depth without (left) or with (right) optical tissue clearing. 2D slices are extracted from indicated depths in the tissue-cleared z-stack to demonstrate image quality at various imaging depths. (**c**) Multiphoton image of 3D global view (2500 μm × 3500 μm × 3000 μm) of optically cleared mouse brain with multiple MDA-MB-231.Br-derived metastases. Red: anti-GFAP; blue: DAPI. (**d**) 2D multiphoton image (top) and 3D surface generated image (bottom) of PNA.Met1 brain metastases stained with anti-cytokeratin 8 (K8). (**e**) 3D multiphoton image of astrocytes (GFAP, red) of the blood brain barrier (left) and astrocyte interaction with MDA-MD-231.Br metastatic cells (right). Blue arrows point to astrocytes, and yellow arrows point to metastatic cells. (**f**) 3D multiphoton image of PNA.Met1 brain metastasis (K8, green) and EdU-tagged nuclei (orange) (top) and enlarged view of dividing nuclei (bottom).

We streamlined a whole tissue clearing, staining, imaging, and computation analysis^17^,^18^ pipeline to quantitatively analyze and thereby enable the elucidation of the functional impact of phenotypic heterogeneity of the metastatic landscape on metastatic outgrowth (Fig. 1a, Supplementary Fig. S1a). The first segment of our pipeline (Fig. 1a, top), consisting of whole tissue clearing, staining, and imaging, ultimately conquers the long-standing challenge of capturing multiple genetic events in their native 3D context *in situ* to allow a holistic view of the tissue and its compositional and spatial heterogeneity. Tissue clearing and refractive index matching rendered the brain lipid-free and optically transparent, allowing for thorough multiplexed molecular phenotyping of large tissue sections (Supplementary Fig. S1b,c and Supplementary Video S1). Further, cleared brain tissue permitted an approximate five-fold increase in imaging depth from ~500 μm (Fig. 1b, left) to ~3000 μm (Fig. 1b, right). Distinguished from previous tissue clearing-based studies, which primarily relied on transgenic mice that express fluorescent proteins, our approach relied on multiplexed staining for proliferative nuclei, metastatic tumor cells, and TME components (e.g., astrocytes), allowing 3D co-registration of multiple metastatic landscape components with high spatial resolution on a global scale of dimensions up to approximately 4000 μm × 4000 μm × 3000 μm (Fig. 1c and Supplementary Video S2). This exponential increase of data content enabled us to reconstruct the brain metastasis landscape in 3D, providing new, exceptionally accurate perspectives on phenotypic heterogeneity, such as the highly irregular tumor morphology that is masked in two-dimensional images (Fig. 1d). We were able to glean detailed information from large, continuous tissue structures, such as blood vessels (Supplementary Fig. S1d), while maintaining high 3D resolution at the cellular level, such as one single extravasated metastatic cell (Fig. 1e), and subcellular details, such as dividing nuclei (Fig. 1f).

### Spatial background removal and forest classifiers-based multi-channel 3D reconstruction (SMART 3D: Spatial Multi-chAnnel ReconstrucTion 3D)

Accurate image segmentation is a prerequisite for quantitative analysis of the spatial relationship between metastatic cells and the metastatic niche. The second and third segments of our integrative pipeline (Fig. 1a, bottom) tackle both the extensive problems (e.g., background removal, multi-channel reconstruction) faced in processing large 3D datasets with multiple channels as discussed in detail below. Despite a significant increase of 3D imaging depth enabled by tissue clearing, strong auto-fluorescence background (noise signal) and inhomogeneous fluorescent staining across the whole tissue significantly limit the applicability of existing 2D computational algorithms for accurate segmentation and visualization of large, multi-channel volumetric 3D datasets. Thus, the first step of our image analysis aimed to remove the background noise in the 3D datasets.

**Figure 2:**
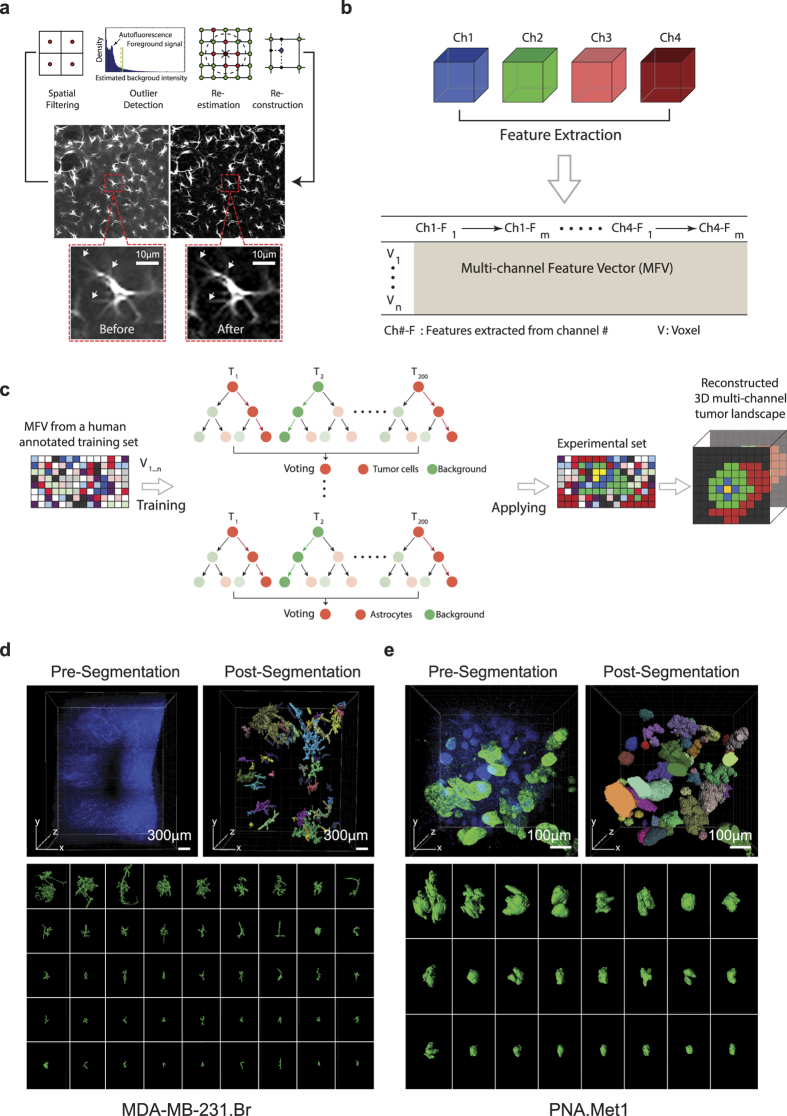
Spatial Filtering-Based Background Removal and Multi-Channel Forest Classifiers-Based 3D (SMART 3D) Reconstruction of Metastatic Heterogeneity. (**a**) Schematic of the background removal process. Arrow points to fine astrocyte processes preserved by this method. (**b**) Schematic of multi-channel feature extraction. (**c**) Schematic of the process of voxel-level segmentation based on random forest classification. (**d**) Image of DAPI stained MDA-MB-231.Br brain metastases sample before (top left) and after (top right) DAPI cluster-based tumor segmentation and surface generation. Color codes represent individually identified tumors. Bottom panel: whole spectrum of individual morphologically heterogeneous tumors. (**e**) Image of DAPI (blue) and K8 (green) stained PNA.Met1 brain metastases before (top left) and after (top right) K8/DAPI-based segmentation and surface generation. Bottom panel: whole spectrum of individual morphologically heterogeneous tumors.

We developed a new approach that combines the spatial filtering method and optimization-based methods for this background removal problem on 3D images^19^. Due to wide variations in the sizes of foreground objects in 3D datasets, it is very difficult to select one single appropriate window size or ball size for the spatial filtering method and the rolling ball algorithm^20^. We applied percentile filtering with a window size that is slightly larger than the size of the smallest object (e.g., the size of a cell) in the image to estimate a rough background. Subsequently, we used unsupervised one-class learning^21^ to detect errors in this rough estimation (Fig. 2a). Finally, these errors were corrected in the re-estimation process and the refined background estimation was obtained for generating a noise-reduced image. The processing speed of our new background removal method^19^ is comparable to the rolling ball algorithm^20^ (~30 hours on a Xenon CPU E5-2660v3 for processing a 512 × 512 dataset). This novel algorithm yields the unprecedented efficiency in removing background while preserving fine structural details (e.g., fine processes of astrocytes) (Fig. 2a, bottom panel).

**Figure 3:**
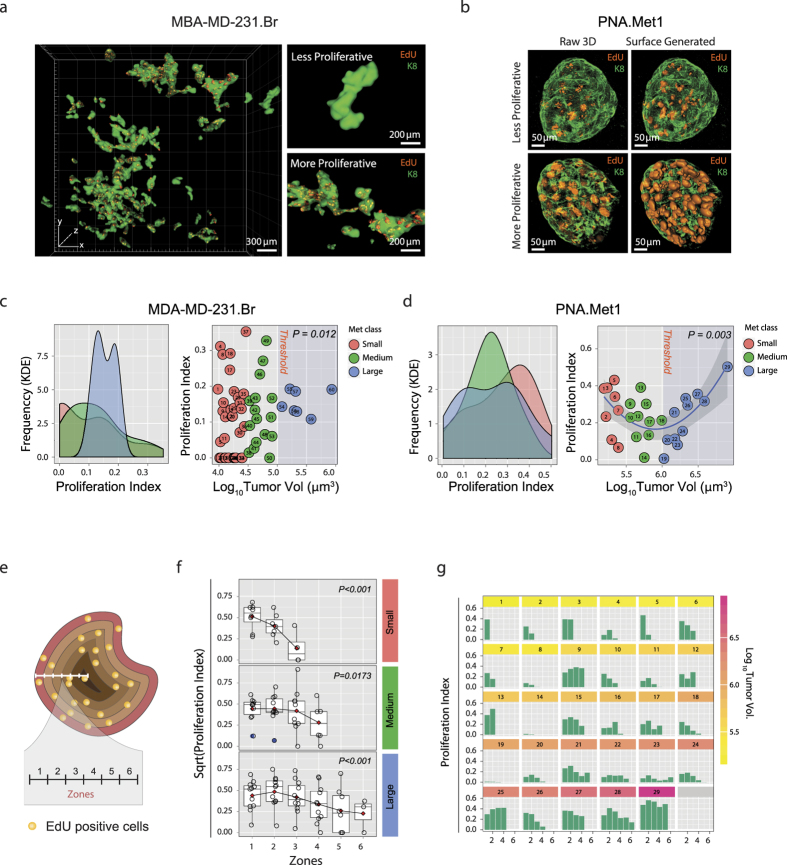
Proliferative Heterogeneity in PNA.Met1 and MDA-MB-231.Br Breast Cancer Brain Metastases. (**a**) Global 3D surface rendering of multiphoton images of MDA-MB-231.Br brain metastases (green) and EdU-tagged nuclei (orange) demonstrating proliferative heterogeneity within the same anatomical region (left) from low proliferation (top right) to high proliferation (bottom right). (**b**) 3D multiphoton images (top) and associated surface generated images (bottom) focusing on EdU-tagged nuclei (orange) in representative less proliferative (left) and more proliferative (right) PNA.Met1 metastases (K8, green). (**c**) Kernel density estimation (KDE) plot of the proliferative indices of small, medium, and large MDA-MB-231.Br metastases (left) and plot of the relationship between the proliferative index and the log_10_ of the tumor volume of each MDA-MB-231.Br metastasis (right). Proliferative index = Total EdU voxel volume/Total tumor voxel volume. (**d**) Kernel density estimation plot of the proliferative indices of small, medium, and large PNA.Met1 metastases (left) and quadratic regression plot of the relationship between the proliferative index and the log_10_ of the tumor volume of each PNA.Met1 metastasis (right). Proliferative index is defined as panel C. (**e**) Schematic representation of concentric zone analysis to analyze spatial characteristics of metastatic proliferation. Zone depth = 10 μm. (**f**) Regression analyses of the proliferative indices among zones of small (top), medium (middle), and large (bottom) PNA.Met1 brain metastases. Red diamond = mean of each group. P value is based on linear regression model. (**g**) Bar graphs of the proliferative index within each 10 μm zone for each PNA.Met1 brain metastasis.

**Figure 4:**
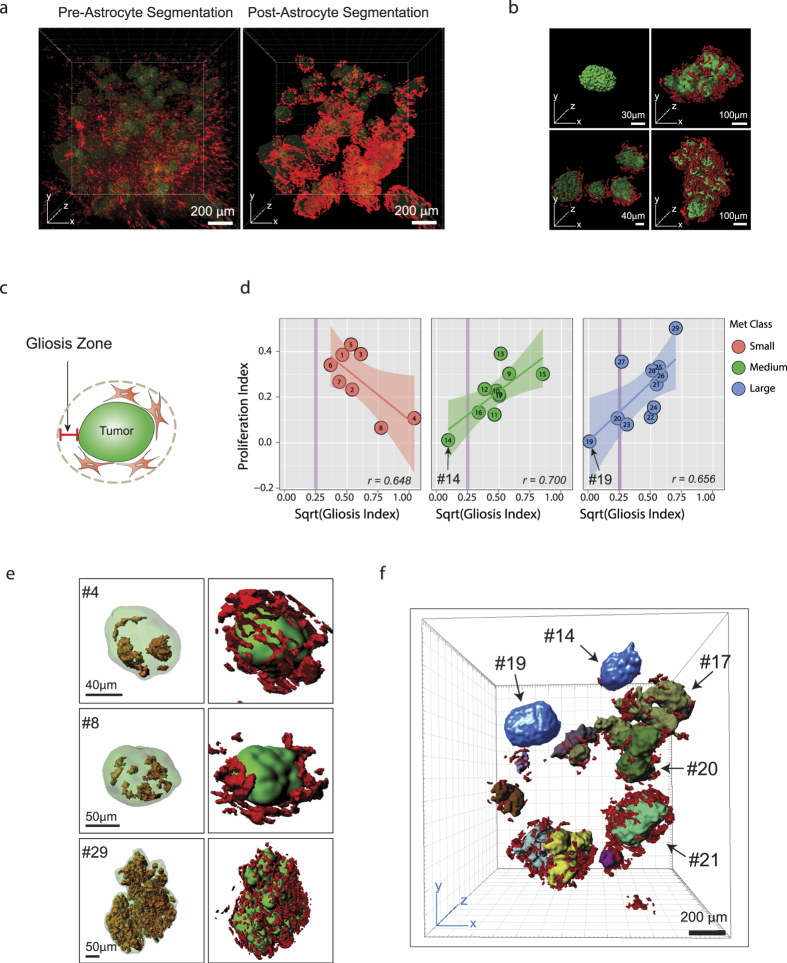
Heterogeneous Astrocyte Response to and Influence on PNA.Met1 Brain Metastases. (**a**) 3D multiphoton image of astrocytes (GFAP+, red) associated with PNA.Met1 brain metastases (K8, green) (left), and corresponding 3D segmentation of the astrocytes (right). (**b**) Representative surface generated images of PNA.Met1 tumors (green) and various levels of associated astrocytes (red). (**c**) Schematic representation of the gliosis zone. Zone depth = 24 μm, the maximum spatial size of one astrocyte. (**d**) Regression plot of the relationship between the proliferative index and the square root (sqrt) of the gliosis index for small (left), medium (middle), and large (right) PNA.Met1 brain metastases. Gliosis index = Total astrocyte voxel volume/Total zone voxel volume. (**e**) Representative surface generated images of PNA.Met1 metastases (green) and EdU-tagged nuclei (orange) (left column) and the metastases and the associated astrocytes (red) (right column). (**f**) Surface generated image of global view of PNA.Met1 metastases (various colors) and associated astrocytes (red), highlighting tumors extremely low gliosis (blue; #14 and #19).

**Figure 5:**
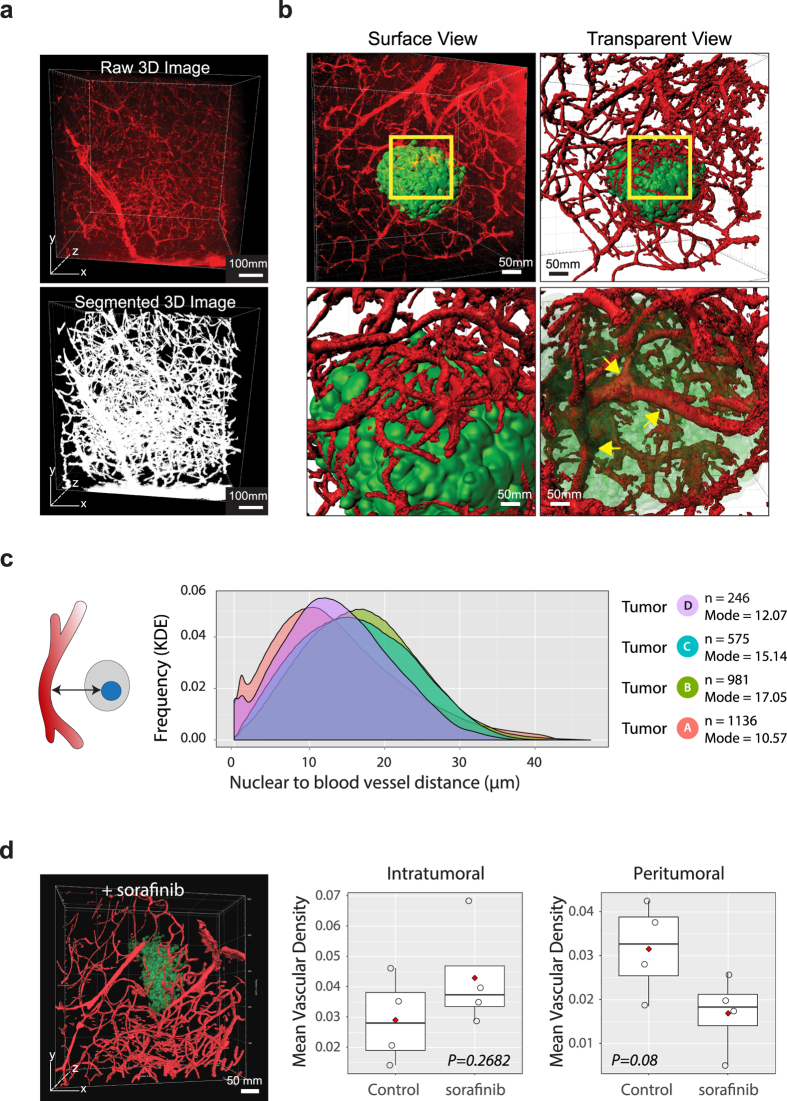
Heterogeneous Spatial and Temporal Vasculature Response to PNA.Met1 Metastases. (**a**) 3D multiphoton image of metastasis-associated vasculature (top) and associated 3D segmentation image (bottom). (**b**) 3D images of metastasis-associated vasculature (red) before (top left) and after (top right) surface generation of vasculature, with a focus on vasculature on metastasis surface (bottom left) and infiltrating vasculature (bottom right). (**c**) Kernel density estimation (KDE) plot for the distance of each tumor cell voxel to the nearest blood vessel for tumors A, B, C, and D. *n*: number of tumor cells. (**e**) Surface generated image of a PNA.Met1 brain metastasis (transparent green) and associated vasculature (red) treated with Sorafenib (left) and plots of the mean vasculature density within the tumor (intratumoral) and outside the tumor (peritumoral) for Sorafenib and control-treated mice. Peritumoral zone = spatial distance within 100 μm from tumor surface.

To compute voxel-level image segmentation with high tolerance of inhomogeneous fluorescent staining, we extracted feature vectors representing each voxel’s appearance and texture from multiple channels and concatenated the feature vectors from different channels into a multi-channel feature vector (MFV) (Fig. 2b). Next, we formulated the voxel-level segmentation problem as a classification problem (Fig. 2c). Several classifiers were trained to determine whether a voxel belongs to a specific type of foreground (e.g., tumor cells, astrocytes, blood vessels, etc.) or the background based on MFV. These classifiers (200 trees and 2 candidate features in each node) were then applied to every voxel in the 3D volumetric datasets to generate the segmentation of desired features (Fig. 2c). To test the accuracy of the classifier, we manually labeled 10 slices for each different component (e.g., tumor, astrocyte, EdU proliferation marker, and blood vessel) and used labeled features as the ground truth. The accuracy was then measured by the F1 score of 10-fold cross validation. F1 score is the harmonic mean of precision and recall, and it is a widely used measure to evaluate the performance of the classifier^22^. According to the F1 score evaluation, volume quantification errors are approximately −3.35%, −0.03%, +8.51%,+1.86% for tumors, astrocytes, proliferating cells, and blood vessels, respectively (Supplementary Table S1). Finally, we applied the SMART 3D pipeline to a series of 3D multi-channel datasets to reconstruct and quantify the metastasis landscape (Figs 2d and 3, 4, 5).

### Heterogeneous morphology of breast cancer brain metastases

To test the robustness of SMART 3D, we generated experimental brain metastases using two breast cancer brain metastasis models with distinct morphologies: MDA-MB-231.brain-seeking (Br) human breast cancer cells in an immunocompromised (Rag1−/−) mouse and PNA.Met1 murine breast carcinoma cells in an FVB host. We stained metastasis-burdened samples with the epithelial cell-specific marker cytokeratin 8 (K8) antibody to identify breast cancer brain metastases, as K8 is absent in normal cerebral cortex (Supplementary Fig. S2a). In addition, tumor cell nuclei displayed unique pleomorphism distinct from neural tissue nuclei, allowing for further nuclear-based tumor cell identification. Our SMART 3D algorithm was adaptive to diverse tumor morphologies in 2D and 3D (Supplementary Fig. S2b,c) and proved to be robust as indicated by the overlap between the original 3D images and the computationally segmented voxels (Supplementary Fig. S2d). Surface renderings of the SMART 3D-segmented metastases within the same metastatic niche revealed a previously underappreciated array of morphological heterogeneity (Fig. 2d,e). The MDA-MB-231.Br metastases are highly branched, reflecting their exclusive growth along brain vasculature (vessel co-option)^23^, whereas PNA.Met1 metastases form irregularly shaped clusters (Supplementary Video S3), reflecting the morphology of brain metastases observed in the clinic. Within the same metastatic niche, the volumes of metastatic tumors are extremely diverse, ranging from 9.6–857.7 × 10^3^ μm^3^ to 155.3–8074.0 × 10^3^ μm^3^ for MDA-MB-231.Br and PNA.Met1 tumors, respectively. This broad spectrum of metastasis volumes suggested a wide range of developmental stages of metastatic lesions within the same TME.

### Metastasis developmental stage shapes a non-linear proliferative heterogeneity

Taking advantage of the multi-channel spatial segmentation and quantification capacities of SMART 3D, we next surveyed the proliferation status of every tumor cell in each metastasis by 5-ethynyl-2′-deoxyuridine (EdU) detection^24^. Both metastasis models displayed a wide spectrum of proliferative indices, from less than 1% to as much as 56%. Interestingly, metastases of similar sizes had very disparate proliferative indices (Fig. 3a,b). Kernel density estimation (KDE, “Gaussian”) for MDA-MB-231.Br metastases showed that small and medium metastases had a wide range of proliferation scattered across proliferative indices (0–0.3) and large metastases had a relatively uniform level of proliferation concentrated between 0.1 and 0.2 proliferative indices. The difference between the variance of proliferation between large metastases (Log_10_ tumor volume >5.0) and small and medium metastases are statistically significant (*p* = 0.012, F-test) (Fig. 3c). KDE (“Gaussian”) for PNA.Met1 metastases revealed a different pattern of proliferation, with a wide range of heterogeneity for small and large metastases, but a narrower range of proliferation for medium metastases primarily concentrated at 0.2 proliferative index (Fig. 3d, left). Interestingly, a parabola-shaped trend (*p* = 0.003, quadratic regression) fit the data of tumor proliferative index versus metastasis volume, in which the vertex of the parabola was within the medium-sized metastasis range and approached a proliferative index of 0.15. Notably, two tumors (i.e., tumors 14 and 19) fell below the vertex and had negligible proliferation (Fig. 3d, right).

Leveraging the spatial aspect of our 3D imaging approach, we next probed the spatial distribution of proliferative cells within metastases. We did so by performing concentric zone analysis in which we analyzed the proliferative indices of concentric 10 μm-thick volumetric zones from the tumor surface to the tumor core (Fig. 3e). Linear regression analysis demonstrated distinct proliferative kinetics in which small tumors have a sharp proliferative index drop-off approaching the tumor core (proliferative index drops from ~0.5 to 0.12, *p* < 0.001), while the tumor core remains proliferative in medium and large sized tumors (Fig. 3f). Notably, there is extensive proliferative heterogeneity both among individual metastases and the zones within individual metastases (Fig. 3g). Even similarly sized tumors have dramatically different maximum proliferative indices (e.g., tumors 18 and 19) and proliferative patterns among zones from the surface to the core (e.g., tumors 16–18).

### Metastasis-induced astrogliosis influences proliferative heterogeneity of tumors

In his “seed and soil” hypothesis, Stephen Paget first proposed that the tumor microenvironment is both essential and important in metastatic seeding and outgrowth^25^. To explore microenvironmental factors that potentially influence metastatic outgrowth while taking advantage of the holistic view provided by 3D imaging, we first examined the astroglial response to brain metastases, a response that 2D imaging insufficiently captures in terms of astrocyte morphology and spatial distribution (Supplementary Fig. S3). Previous studies have suggested that the astroglial response promotes metastatic outgrowth^26^,^27^,^28^,^29^,^30^,^31^,^32^,^33^. To accurately characterize the astrocyte response to brain metastases, we imaged the astroglial response in 3D to obtain complete astrocyte morphology in exquisite detail (Supplementary Fig. S3) and further applied SMART 3D to clearly distinguish anti-GFAP staining from background noise in 2D and 3D (Supplementary Fig. S3). This enabled us to reconstruct and quantitatively analyze spatial astrogliosis patterns on a global scale (Fig. 4a). Qualitative analysis of astrogliosis revealed that astrocyte coverage of tumors was not spatially uniform around metastases and the volume of astrogliosis was variable among metastases in both the PNA.Met1 model (Fig. 4b) and the MDA-MB-231.Br model (Supplementary Fig. S4). Notably, the early astroglial response (gliosis index) appeared to be negatively correlated with metastasis proliferation (*r* = *0.648*) (Fig. 4d). When metastatic outgrowth progressed, the level of astrogliosis was positively correlated with the proliferation indices of metastases in both medium (*r* = *0.700*) and large (*r* = *0.656*) tumor groups (Fig. 4d,e). Interestingly, two medium to large-sized metastases (i.e., tumors 14 and 19) have negligible gliosis, and also have extremely low proliferative indices. Importantly, tumor 19, which had no associated astrogliosis, was not proliferative, whereas similarly sized tumors (i.e., tumors 17, 20, and 21) within the same metastatic niche as tumor 19 were surrounded with reactive astrocytes and thrived with active proliferation (Fig. 4f), suggesting a role of astrogliosis in sustaining metastasis growth. Unlike PNA.Met1 metastases, global analyses of MDA-MB-231.Br metastases showed no clear correlation between gliosis index, proliferation index, or metastasis size (Supplementary Fig. S4), indicating a non-essential role of astrogliosis in MDA-MB-231.Br brain metastases. It is likely that the exclusive growth of these metastases along blood vessels (Supplementary Fig. S4a) provides these metastases with a direct nutrient supply that is the ultimate determinant of their outgrowth.

### Heterogeneous spatiotemporal angiogenic response to brain metastases

Patterns of brain tumor and brain metastasis angiogenesis are highly debated^34^,^35^,^36^ primarily due to a lack of approaches that can capture blood vessels in their entirety *in situ*. By taking advantage of our tissue clearing-based imaging approach and SMART 3D analysis pipeline, we explored the global intratumoral and peritumoral angiogenesis patterns in 3D (Fig. 5a). Qualitative analyses showed extensive, tortuous tumor-associated vasculature comparable to previous experimental and clinical observations^37^,^38^,^39^. We also observed highly branched vessels covering the tumor surfaces and few but large infiltrating blood vessels inside the tumors, which were previously underappreciated features (Fig. 5b, Supplementary Fig. S5a, Supplementary Video S4). To reveal the global spatial relationship of each tumor cell to the surrounding blood vessels, we further quantified the spatial distance from each tumor cell voxel to the nearest blood vessel (Fig. 5c). Although metastatic tumors analyzed contain different numbers of tumor cells (*n* = 264 for tumor D and *n* = 1136 for tumor A), as measured by the mode frequency of density curve, most tumor cells were located less than 40 μm away from the nearest blood vessel, a pattern consistent among all metastasis sizes, which is significantly closer than the previously documented limit (100 μm) derived from 2D histology studies^40^.

To further examine the spatial patterns of angiogenesis, we performed concentric zone analysis covering zones from the core of metastases to the closest vessel outside the metastases (Supplementary Fig. 5b,c). Despite different tumor sizes, the maximum relative vascularisation index inside each tumor is approximately 0.02. The vascularisation index of smallest tumor (Tumor D) progressively declined from the tumor surface to the core. Tumor C had an increase of relative vasculature volume approaching the middle zone, after which point the relative vasculature volume sharply decreased, producing an inverted parabola, implying *de novo* angiogenesis inside the tumor.

Finally, to test the whether the SMART 3D-based angiogenesis analysis could potentially be used to evaluate anti-angiogenic therapies, we treated brain metastasis-bearing mice with multi-kinase inhibitor Sorafenib, a FDA approved small molecule inhibitor which has been shown to be blood brain permeable and enter the brain^41^ and exhibit encouraging efficacy in treating brain metastases derived from renal cell carcinoma^42^. Surprisingly, despite a discernable decline of peritumoral angiogenesis, the intratumoral mean vascular density (MVD) did not significantly decrease (Fig. 5d).

## Discussion

Metastatic colonization and outgrowth are believed to be spatially heterogeneous and temporally dynamic. While current state-of-the-art single-cell sequencing technologies have made strides toward understanding the genetic basis of tumor heterogeneity, deciphering the spatial and compositional heterogeneity of metastasis remains heavily reliant on conventional 2D histology. It is imperative to develop and integrate novel techniques from multiple disciplines to explore metastasis from spatial perspectives. In this study, we sought to do so by further developing and integrating cutting-edge technologies from the fields of both neuroscience and computational science. Our unique, integrative approach combining whole tissue clearing, staining, and imaging, with computer-assisted segmentation and quantifications (SMART 3D) provided us leverage in studying the metastatic landscape with unparalleled accuracy. Specifically, we have demonstrated the power of whole tissue imaging in delineating astonishing morphological diversity of metastatic tumors. More importantly, we demonstrated the feasibility of using multiplexed immunostaining to molecularly characterize the spatially heterogeneous metastasis landscape. With further refinement of whole tissue staining procedures and validation of antibodies for cleared tissue, we expect multiplexed molecular staining will produce unprecedented mechanistic insight into many biological phenomena in the near future.

With the maturation of tissue clearing techniques, using this whole tissue imaging approach to explore tissue structure or tumor heterogeneity becomes highly desirable for many laboratories. However, quantitative analysis of 3D volumetric datasets that are terabytes in size imposes new, significant challenges. Our study provides one possible solution (SMART 3D) for quantitative analysis of 3D, multi-channel volumetric data. First, by developing and applying a spatial background removal algorithm, we effectively removed inhomogeneous fluorescent background present in most biological samples and further induced by immunostaining. Effective background removal in 3D images forms the foundation for downstream segmentation and quantification. Second, we constructed a machine learning (forest classifiers) based multi-channel 3D reconstruction pipeline by integrating biologically meaningful factors annotated by biologists and computational features intrinsic to 3D volumetric datasets. In our experience, applying this machine-learning pipeline is critical to more effectively extract and recover structural information from large 3D volumetric data that inevitably varies significantly within the same dataset. Lastly, our study demonstrated how our integrative whole tissue imaging and analysis platform could help biologists generate statistically significant correlations and discern previously unknown patterns that imply novel hypotheses of molecular mechanisms to be formulated and further explored.

Our study applying our integrative pipeline revealed a previously underappreciated heterogeneity in the metastatic landscape, and our spatial analysis of the metastasis landscape also shed light on how the TME impacts metastatic outgrowth. Two examples of such microenvironmental factors/processes we investigated in our study are astrocytes (in the process of astrogliosis) and vasculature. Through cross-referencing metastasis proliferation, tumor developmental stage, and astrogliosis, we revealed a dual, stage-dependent role of astrocytes on metastatic outgrowth (Fig. 4). Through global proliferation analysis coupled with spatially defined blood vessel density, we noted that the majority of cells in metastatic tumors are located in close proximity (between 10–20 μm) to blood vessels (Fig. 5c). More interestingly, the anti-angiogenic treatments led to a divergent spatial pattern of blood vessel distribution, which should be closely examined in the future studies. Collectively, results from our unbiased global approach further underscore a critical role of metastatic niche environmental factors and their potential role in sustaining metastatic outgrowth or metastatic dormancy. These observations will lead to new avenues for further functional investigations of novel mechanisms of metastasis.

With the continuous development of new, better tissue clearing methods and optics technology, our platform and SMART 3D will only grow in relevance. Our platform is designed to be able to integrate any given tissue clearing method, and SMART 3D is capable of processing and analyzing image data sets derived from a variety of 3D, multi-channel high content imaging strategies. In this study, we demonstrated the ability of SMART 3D to adapt to analyzing datasets derived from the most recently developed tissue clearing methods, including CUBIC 2, PACT, and Sca*l*eAB^43^,^44^,^45^. Furthermore, our platform will become even more vital for researchers applying new tissue clearing methods, which are increasingly compatible with multiple antibodies (Supplementary Table S2). Increasing ability to simultaneously stain for multiple markers will dictate the use of several channels during image acquisition, necessitating the multi-channel processing and analysis of which our platform is uniquely capable. Advanced optics systems for imaging will decrease image acquisition time while increasing the data content obtained, which SMART 3D will be able to process and analyze. Our integrative pipeline, unlike most methods and technologies, promises to become increasingly relevant with time and provide a scaffold by which researchers can tackle complex biological questions.

Taken together, we developed an integrative platform for 3D quantitative analysis of the spatially and compositionally heterogeneous metastasis landscape. Using whole tissue multiplexed staining and fluorescence imaging coupled with our SMART 3D image analysis pipeline, we demonstrated intriguing patterns of spatial heterogeneity of the metastatic landscape. By integrating multidisciplinary expertise, our proof-of-concept study, the first of this type, further demonstrates the necessity of examining metastases in 3D *in situ*, the practicality of this approach, and the novel concepts and discoveries that may be derived from 3D quantitative image analysis.

## Methods

### Cell Culture

The parental MDA-MB-231 cells were purchased from ATCC. MDA-MB-231.Br cell line was developed by *in vivo* selection of brain tropic derivatives for a minimum of three rounds through intra-cardic injection. PNA.Met1 cell lines were established from spontaneous primary murine mammary tumors from MMTV-PyMT transgenic mouse (FVB background). MDA-MB-231.Br cell line was cultured in DMEM F12 medium supplemented with 10% fetal bovine serum (FBS) and 1% penicillin streptomycin (Pen-Strep). PNA.Met1 cells were cultured in DMEM high glucose medium supplemented with 10% FBS and 1% Pen-Strep. Both cell lines were maintained at 37 °C in a 5% CO_2_ humidified environment and subcultured upon reaching approximately 85% confluence. Immediately prior to injection, cells were rinsed three times with 1 × PBS, trypsinized, and pelleted twice and resuspended in serum-free RPMI medium to a concentration of approximately two million cells/mL.

### Brain Metastasis Mouse Models and *In Vivo* Experiments

All animal experiments were performed ethically and in accordance with IACUC protocol approved by the University of Notre Dame IACUC committee. FVB and congenic Rag1−/− (C.129S7(B6)-Rag1^tm1Mom^/J) mice were purchased from The Jackson Laboratory (Bar Harbor, ME). All mice were eight weeks or older prior to experimental procedures. *In vivo* brain metastases were formed by either injection of 100 *μ*L (~200,000 cells) cell suspension via the right internal carotid artery or intra-cranial injection of 690 nL of ~70,000 cells (drug treatment experiments). Prior to and during injection of cancer cells, mice were anesthetized with isoflurane. Following surgery, mice received 100 *μ*L subcutaneous injections of Baytril^®^ (2.27%, Bayer HealthCare LLC, Animal Health Division), Ketoprofen^®^ (1 mg/mL) and 1mL of 0.9% NaCl. FVB mice injected with PNA.Met1 cells were sacrificed after two weeks following cell injection, while the Rag1−/− mice injected with MDA-MB-231Br cells were sacrificed between three to four weeks after injection. Two hours prior to sacrifice, mice were injected with 100 *μ*L of 100 mg/mL 5-ethynyl-2′-deoxyuridine (EdU, Life Technologies, Cat. No. 10639) via tail vein. For labeling blood vasculature, animals were injected with 100 *μ*L 10,000 MW Dextran, Alexa Fluor^®^ 488 or Alexa Fluor^®^ 594 (Life Technologies™, Cat. No. D-22910 or D-22913) via tail vein five minutes prior to sacrifice when the brain was intended to be put through PACT or CUBIC tissue clearing. In instances when the brain was intended to be cleared by the ScaleAB protocol, cardiac perfusion with 10mL or DiR (Sigma, Cat. No. 43608) was performed as described previously^46^. Immediately prior to and during sacrificing, mice were anesthetized with isoflurane. Mice that did not receive a Dextran injection or were not perfused with DiR were perfused transcardially with 10 mL chilled 1× PBS immediately followed by 10 mL chilled 4% paraformaldehyde (PFA). Mice that did receive a Dextran injection were not perfused. Brains were extracted, cut in half sagittally, and placed in 4% PFA for 24 hours at 4 °C with gentle rocking. For anti-angiogenic treatment experiments, Sorafenib (LC Laboratories, Woburn, MA) was administered to mice at a concentration of 50 mg/kg by intra-peritoneal injection once daily 10 days following metastasis induction. Sorafinib was diluted in Cremophore EL (Sigma

### Tissue Clearing and Staining

Slightly modified CUBIC and PACT tissue clearing protocols^43^,^47^ were used as follows for brain tissue clearing, with the exception of the Sorafinib treated brains. Following 24 hours fixation, brains were rinsed twice with 1 × PBS, sliced into 2 mm sagittal sections, and incubated in a hydrogel formulation (2 or 4% acrylamide in 1x PBS with 0.25% photoinitiator VA044 [Wako Chemicals]) at 4 °C gently rocking for three days. Prior to hydrogel polymerization, samples were degassed using a desiccation chamber, alternating between three 10-minute cycles of vacuum and nitrogen gas. Samples were polymerized by incubation at 37 °C for 3–4 hours. Polymerized hydrogel was decanted and samples were rinsed twice with 1 × PBS. Lipid removal from samples was then performed using either 8% SDS in 1 × PBS. Once samples became optically transparent after approximately 4–7 days, samples were washed for 24 hours in 1 × PBS before whole tissue staining (molecular phenotyping). For molecular phenotyping, all samples were first incubated in a 1:50 primary antibody dilution (anti-Ms mAb GFAP (GA5), Cell Signaling Technology Technologies^®^, Cat. No. 3670s; anti-Rb mAb Cytokeratin 8 (EP1928Y), Abcam^®^, Cat. No. ab53280) in 0.1% Triton X-100 for 3 to 7 days with gentle rotating. Following primary antibody incubation, samples were washed for 24 hours in 0.1% Triton X-100 with minimum three rounds of buffer changes. All samples were then incubated in a 1:50 secondary antibody (goat anti-Rb or goat anti-Ms Alexa Fluor^®^ 488, 594, or 647) dilution in 0.1% Triton X-100 for 3 to 7 days with gentle rotating. After secondary antibody incubation, EdU was detected per Click-iT EdU Alexa Fluor^®^ 488 or 594 imaging kit (Life Technologies™, Cat. No. 10639) protocol with a 3–4 hour detection incubation period. Some samples were incubated in DAPI (0.025 mg/mL) for 12 hours with gentle rotating. Samples were washed one last time in 0.1% Triton X-100 for 24 hours with multiple buffer changes and then made optically transparent by incubation in CUBIC 2 reagent prepared as described previously^43^ for at least 2 hours prior to imaging. The brains of Sorafinib treated mice were cleared using ScaleAB methodology as previously described^44^.

### Confocal and Two-Photon Microscopy

All images were acquired using either the confocal or two photon setting on a commercial multiphoton laser scanning inverted microscope (Olympus FV1000) equipped with filter set (460–500, 520–560, 525–625, 650–700 nm) and a mode-locked Ti:sapphire laser (Mai Tai DeepSee 690–1040 nm, Spectra-Physics). Optical cleared and stained samples were immersed in CUBIC 2 reagent in a custom-made sample holder when imaged using a 10× ScaleView objective (XLPLN10XSVMP, Olympus USA; NA = 0.6 and WD = 8 mm) or placed on a coverslip when imaged using the 25× objective (XLSLPLN25XGMP, Olympus USA; NA = 1.0 and WD = 8 mm). Use of confocal versus multiphoton was dictated by which method better excited the Alexa fluorophores, which depended on fluorophore vendor and imaging depth. When acquiring images to be stitched, MATL was used in the Olympus software to program 10% overlap between imaging stacks.

### Image Processing and Segmentation

#### Background removal

Auto-fluorescence in mouse brains results in a strong and inhomogeneous background in the acquired 3D images. Such inhomogeneous background (noise) causes difficulties to segmentation and visualization of the 3D images. We developed a new approach^19^ that combines the spatial filtering method and optimization-based methods for this background removal problem. First, we applied percentile filtering with a window size that is slightly bigger than the size of the smallest object (e.g., the size of a cell) in the image, to estimate a rough background. Then, we used unsupervised one-class learning^21^ to detect errors in this rough estimation. Finally, these errors were corrected in the re-estimation process and the refined background estimation was obtained for generating a noise-free image. The processing speed of our new background removal method^19^ is comparable to the rolling ball algorithm^20^ and yields the state-of-the-art accuracy. More details and validations can be found in^19^.

#### Voxel-level segmentation

We utilized information from multiple channels (e.g. K8, DAPI, GFAP, and EdU) for segmentation. For this purpose, we formulated the voxel-level segmentation problem as a classification problem. Several classifiers were trained to determine whether a voxel belongs to a specific type of foreground (tumor cells, astrocytes, blood vessels, etc) or the background based on multi-channel features. These classifiers were then applied to every voxel in the 3D image to generate the segmentation.

(1) *Multi-channel feature extraction*: The classification accuracy relies heavily on the quality of the extracted features. In order to utilize multi-channel information, our multi-channel feature extraction procedure for the voxels consists of two main steps. (i) Extracting a feature vector in each channel. As in^48^, the following features are extracted for every voxel in each channel to represent its appearance and texture: Intensity, gradient magnitude, eigenvalues of the Hessian matrix, and eigenvalues of structure tensor; (ii) concatenating together feature vectors from different channels to form a multi-channel feature vector. (2) *Voxel classification*: After the feature extraction process, training data were labeled by human experts and used to train four random forest classifiers^49^ that were designed to classify tumor cells, astrocytes, proliferating cells, and blood vessels. Each classifier has 200 trees and 2 candidate features in each node. These classifiers are then applied to every voxel in the 3D image to generate segmentation results.

#### Structural-level segmentation

Since tumor cells tend to form dense clusters, to eliminate false-positives, cell clusters whose volumes are larger than a threshold in the voxel-level segmentation of tumor cells were identified as tumor clusters. The volume of each cell cluster was approximated by the volume of its connected component in the voxel-level segmentation. The threshold for large clusters was selected by a human expert for each 3D image. When K8 staining was used, each cell cluster that contains K8 signal was immediately identified as a tumor cluster regardless of its size.

### Image Quantification

Based on the structural-level segmentation and voxel-level segmentation, we performed the following quantifications. (1) *Proliferation ratio:* By assuming that every cell of a specific type has an average volume, the proliferation ratio of each tumor cluster can be determined approximately as the ratio of the volume of proliferating cells inside the tumor to the volume of the tumor cluster. The volume of proliferating cells was computed based on their voxel-level segmentation and the volume of the tumor cluster was computed as describe above. (2) *Volume of surrounding astrocytes (astrogliosis)*: For each tumor cluster, we define its surrounding space as consisting of all the voxels that satisfy the following two conditions: (a) It is outside the tumor cluster, and (b) its closest distance to the tumor surface is smaller than a chosen value *dis* (the average diameter of an astrocyte is 24 μm. Therefore we set *dis* = 24 μm to survey the single layer of astrocytes surrounding the tumor for astrocytes). The surrounding space was calculated in two steps: (i) dilate the cluster region in the original image in a spherical manner with a radius *dis*; (ii) subtract the original cluster region from the dilated region. Then the volume of the surrounding astrocytes can be easily calculated. (3) *Zone analysis*: Zone analysis was performed in a similar manner. Each zone was calculated using image morphological or logical operations. (4) *Distribution of the closest distances from tumor cells to blood vessels*: We computed approximately the distribution of the closest distances from tumor cells to blood vessels by computing the distribution of the closest distances from tumor cell voxels to blood vessel voxels, which was carried out by using the k-nearest neighbor search function in MATLAB.

### 3D Volumetric Data Presentation

3D images were obtained by stacking up and aligning 2D image slices based on minimizing the mean squared error (MSE) between aligned regions in consecutive slices using a custom MATLAB code and XUVStitch (http://www.xuvtools.org). All surface generated images and videos of original data or surface generated data were created using IMARIS software (Bitplane).

Raw quantification data were extracted by using MATLAB, and statistical analysis was performed in R studio. *P* < 0.05 (two-tailed) was considered statistically significant.

## Additional Information

**How to cite this article**: Guldner, I. H. *et al.* An Integrative Platform for Three-dimensional Quantitative Analysis of Spatially Heterogeneous Metastasis Landscapes. *Sci. Rep.* **6**, 24201; doi: 10.1038/srep24201 (2016).

## Supplementary Material

Supplementary Information

Supplementary Video S1

Supplementary Video S2

Supplementary Video S3

Supplementary Video S4
