# Supplementary material for: An Integrative Platform for Three-dimensional Quantitative Analysis of Spatially Heterogeneous Metastasis Landscapes
Source: Sci Rep. 2016 Apr 12;6:24201. doi: 10.1038/srep24201 (PMC4828720; doi:10.1038/srep24201)

Supplementary Information

Title: Integrated Platform for Three-dimensional Quantitative Analysis of Spatially Heterogeneous Metastasis Landscape

Ian H. Guldner[1,4], Lin Yang[2], Kyle R. Cowdrick[1,4], Qingfei Wang[1,4], Wendy V. Alvarez Barrios[1,4], Victoria R. Zellmer[1,4], Yizhe Zhang[2], Misha Host[1,4], Fang Liu[3,4], Danny Ziyi Chen[2,4], Siyuan Zhang[1,4, *]

Affiliations of authors:

[1] Department of Biological Sciences, College of Science, University of Notre Dame, Notre Dame, IN 46556, USA

[2] Department of Computer Science and Engineering, College of Engineering, University of Notre Dame, Notre Dame, IN 46556, USA

[3] Department of Applied and Computational Mathematics and Statistics, College of Science, University of Notre Dame, Notre Dame, IN 46556, USA

[4] Mike and Josie Harper Cancer Research Institute, University of Notre Dame, 1234 N. Notre Dame Avenue, South Bend, IN 46617, USA

[#] These authors contributed equally to this work.

*** Correspondence to:** Siyuan Zhang, M.D., Ph.D., Department of Biological Sciences, University of Notre Dame, A130 Harper Hall, Notre Dame, IN 46556. E-mail: szhang8@nd.edu; Telephone: 713-792-3636; Fax: 713-792-4454.

Supplementary Legends

Supplementary Figure S1 | Tissue optical clearing based imaging pipeline for molecular phenotyping of brain metastasis. (a) Schematic for the timeline of the experimental pipeline. **(b)** Brain tissue slice before (top) and after (bottom) tissue clearing and refractive index matching. **(c)** 3D multiphoton raw image (top) and surface generated image (bottom) demonstrating multiplexed staining for metastases (K8, green), EdU-tagged nuclei (orange), and astrocytes (GFAP, red). **(d)** 3D surface generated image of highly branched vasculature in mouse brain (red).

Supplementary Figure S2 | Metastasis segmentation based on tumor specific staining and nuclear of tumor cells. (a) Image of anti-cytokeratin K8 staining (K8, red) detected in leptomeninges of normal brain. Left DAPI stain (blue), middle K8 stain, right merge. **(b)** 2D overlay of K8 and DAPI (colored images; K8, green; DAPI, blue) and corresponding DAPI/K8 segmented images (white) in two metastasis models. **(c)** 3D DAPI staining and overlay of K8 and DAPI (colored images; K8, green; DAPI, blue) and corresponding DAPI/K8 segmented images (white) in two metastasis models. **(d)** 3D multiphoton image of DAPI (blue) and K8 (green) stained PNA.Met1 metastases (left), corresponding segmented 3D image (middle), and overlap of original image and segmented data demonstrating nearness of segmentation to the ground truth (right).

Supplementary Figure S3 | 3D segmentation of astrocytes. (a) Schematic representing the incomplete view of astrocytes obtained by 2D imaging (left) and a 2D multiphoton image of

astrocytes (GFAP, red). **(b)** 3D multiphoton images captures gross astrocyte morphology, including fine protrusions. **(c)** 2D segmentation of astrocytes.

Supplementary Figure S4 | Heterogeneous Astrocyte Response to MDA.MB.231br Brain Metastases. (a)3D surface generated image of global MDA-MB-231.Br brain metastases (green) and associated astrocytes (red) (left), demonstrating high gliosis levels (top right) and low gliosis levels (bottom right). **(b)** Plot of relationship between the proliferative index and the square root (Sqrt) of the gliosis index for small (left), medium (middle), and large (right) MDA-MB-231.Br brain metastases.

Supplementary Figure S5 | 3D reconstructed view of metastasis associated vasculature. (a) Representative surface generated images global (left), surface (middle), and intratumoral (right) metastasis-associate vasculature in tumor B and tumor D. **(b)** Schematic of concentric zone analysis of intratumoral and peritumoral vascularization index. **(c)** Plots of vascularization index per intratumoral zones (negative numbers) and peritumoral zones (positive numbers) for metastases A, B, C, and D. Vascularization index = Total vasculature voxel volume / Total zone voxel volume.

Supplementary Video S1 | Global View of MDA-MB-231.Br Brain Metastasis Landscape. 3D view of MDA-MB-231.Br brain metastases identified by DAPI (blue) clustering and astrocytes (GFAP, orange) demonstrating both the global view achieved with the simultaneous ability to reach single cell resolution.

Supplementary Video S2 | Multiplexed Staining of MDA-MB-231.Br Brain Metastasis Landscape. 3D view of MDA-MB-231.Br brain metastasis landscape with multiplexed staining for brain metastases (K8, green), nuclei (DAPI, blue), EdU-tagged nuclei (EdU, purple), and astrocytes (GFAP, orange). Brain metastasis staining reveals highly irregular shape of MDA-MB-231.Br brain metastases and proliferative heterogeneity.

Supplementary Video S3 | Multiplexed Staining of PNA.Met1 Brain Metastasis Landscape. 3D view of PNA.Met1 brain metastasis landscape with multiplexed staining for brain metastases (K8, green), EdU-tagged nuclei (EdU, orange), and astrocytes (GFAP, red). Brain metastasis staining reveals highly irregular shape of PNA.Met1 brain metastases and proliferative heterogeneity.

Supplementary Video S4 | 3D View of PNA.Met1 Metastasis-Associated Vasculature. 3D surface generation of metastasis-associated vasculature (red) shows think, highly branched vessels on the metastasis surface and few, but large blood vessels infiltrating the tumor.

Supplementary Table 1 Performance evaluation of the imaging segmentation classifier

	Tumor	Astrocytes	Proliferation	Vessels
Precision	0.9698	0.9352	0.9200	0.9333
Recall	0.9373	0.9349	0.9983	0.9507
Accuracy	0.9963	0.9758	0.9971	0.9980
F1 Score	0.9532	0.9350	0.9575	0.9417

<u>**Note:**</u> The F1 score is the harmonic mean of precision and recall and is a widely used measure to evaluate the performance of the classifier. The error of the volume quantification can be calculated by the following equation:

$$Error_{volume} = \frac{Calculated\ Volume}{True\ Volume} - 1 = \frac{TP + FP}{TP + FN} - 1 = \frac{Recall}{Precision} - 1$$

In this equation, TP denotes true positive, FP denotes false positive, and FN denotes false negative. According to the evaluation, the error of the tumor volume quantification is around -3.35%, the error of the astrocytes volume quantification is around -0.03%, the error of proliferating cells volume quantification is around +8.51%, and the error of the blood vessel volume quantification is around +1.86%.

The error of the ratio quantification can be calculated by the following equation:

$$Error_{ratio} = \frac{Calculated\ Ratio}{True\ Ratio} - 1$$

$$= \frac{Calculated\ volume_{other\ component}/Calculated\ volume_{tumor}}{True\ volume_{other\ component}/True\ volume_{tumor}} - 1$$

$$= \frac{1 + Error_{volume\ other\ component}}{1 + Error_{volume\ tumor}} - 1$$

The error of the tumor-astrocyte ratio quantification is around +3.44%, the error of the tumor-proliferating cells ratio quantification is around +12.27%, and the error of the tumor-vessel ratio quantification is around +5.39%.

Supplementary Table 2 Dyes, Stains, and Antibodies Compatible with Various Tissue Clearing Methods

Dye/antibody	Source	Application	SMART 3D compatibility	Reference
DAPI	Life Technologies (D1306)	nuclear staining	Yes	SMART3D
Hoechst	Life Technologies (H3570)	nuclear staining	Yes	SMART3D
Dil/DiO/DiR	Life Technologies (V-22889)	membrane staining	Yes (with Scale S)	SMART3D
EdU	Life Technologies (C10339)	proliferation marker	Yes	SMART3D
mouse anti-GFAP	CST (3670S)	astrocyte marker	Yes	SMART3D
rabbit anti-GFAP	CST (12389)	astrocyte marker	Yes	SMART3D
rabbit anti-cytokeratin 8	Abcam (ab53280)	epithelial cell marker	Yes	SMART3D
dextran, fixable, Alexa-fluor conjugated	Life Technology (D22914)	pseudo-blood vessel marker	Yes	SMART3D
Parvalbumin	Abcam	neuron marker	Not Tested	CLARITY[1]
Tyrosine Hydroxylase	Abcam	neuron marker	Not Tested	CLARITY[1]
Propidium Iodide	Life Technology	nuclear staining	Yes	CLARITY[1]
anti-Neurofilament NF-H	Aves	neuron marker	Not Tested	CLARITY[1]
anti-GFP	Invitrogen	GFP	Not Tested	CLARITY[1]
mouse anti-α-smooth muscle actin	Sigma (C6198)	vascular structure marker	Not Tested	CUBIC[2]
mouse anti-Pan cytokeratin antibody	Abcam (ab78478)	epithelial cell marker	Not Tested	CUBIC[2]
chicken anti-tyrosine hydroxylase	Aves	neuron marker	Not Tested	PACT[3]
rabbit anti-integrin b4	Santa Cruz	general cellular staining	Not Tested	PACT[3]
rabbit anti-b5 IgG	Santa Cruz	general cellular staining	Not Tested	PACT[3]
rabbit anti-beta tubulin IgG	Santa Cruz	beta tubulin marker	Not Tested	PACT[3]
NeuroTrace 530 / 615 Red Fluorescent Nissl Stain	Life Technology (N-21482)	neuron marker	Yes	PACT[3]
mouse anti-amyloid-β	Covance (SIG-39347)	amyloid plaque makerer	Not Tested	ScaleS[4]

rabbit anti-NeuN	Millipore (ABN78C3)	neuron marker		ScaleS[4]
rabbit anti-Iba1	Wako (019-19741)	macrophage/microglia marker	Yes	ScaleS[4]
goat anti-TrkC	R & D Systems (AF1404)	DRG neuron marker	Not Tested	iDISCO[5]
goat anti-mouse TrkB	R & D Systems (AF1494)	DRG neuron marker	Not Tested	iDISCO[5]
goat anti-human ROBO3	R & D Systems (AF3076)	roundabout homolog 3	Not Tested	iDISCO[5]
goat anti-mouse Ret	R & D Systems (AF482)	DRG neuron marker	Not Tested	iDISCO[5]
goat anti-choline aceytltransferase	Millipore (AB114P)	cholinergic neuron marker	Not Tested	iDISCO[5]
anti- tyrosine hydroxylase	Millipore (AB152)	dopaminergic neuron marker	Not Tested	iDISCO[5]
guinea pig anti-VGLUT2	Millipore (AB2251)	vesicular glutamate transporter 2	Not Tested	iDISCO[5]
mouse anti-neuN	Millipore (MAB377)	neuronal nuclei	Not Tested	iDISCO[5]
rabbit anti-c-Fos	Santa Cruz (sc-52)	neuron activity marker	Not Tested	iDISCO[5]
goat anti-AQP2	Santa Cruz (sc-9882)	kidney collection duct marker	Not Tested	iDISCO[5]
rabbit anti-E-cadherin	CST (3195)	intestinal microvillosities marker	Not Tested	iDISCO[5]
anti-cleaved caspase 9	CST (9509)	apoptotic cell marker	Not Tested	iDISCO[5]
anti-cleaved caspase 3	CST (9661)	apoptotic cell marker	Not Tested	iDISCO[5]
rabbit anti-N cadherin	Abcam (ab12221)	cadherin-2	Not Tested	iDISCO[5]
rabbit anti-FOXP2	Abcam (ab16046)	developing neuron marker	Not Tested	iDISCO[5]
rabbit anti-histone H3	Abcam (ab5176)	mitotic cell maker	Not Tested	iDISCO[5]
rabbit anti-laminin	Sigma-Aldrich (L9393)	kidney tubule maker	Not Tested	iDISCO[5]
rat anti-mouse CD31	BD Bio (550274)	vasculature marker	Not Tested	iDISCO[5]
rabbit anti-GFP	Life Technology (CAB4211)	GFP	Yes	iDISCO[5]

Reference cited:

1. Chung, K. *et al.* Structural and molecular interrogation of intact biological systems. *Nature* **497,** 332–337 (2013).
2. Tainaka, K. *et al.* Whole-body imaging with single-cell resolution by tissue decolorization. *Cell* **159,** 911–924 (2014).
3. Yang, B. *et al.* Single-cell phenotyping within transparent intact tissue through whole-body clearing. *Cell* **158,** 945–958 (2014).
4. Hama, H. *et al.* ScaleS: an optical clearing palette for biological imaging. *Nat. Neurosci.* **18,** 1518–1529 (2015).
5. Renier, N. *et al.* iDISCO: A Simple, Rapid Method to Immunolabel Large Tissue Samples for Volume Imaging. *Cell* **159,** 896–910 (2014).

Supplementary Figure 1

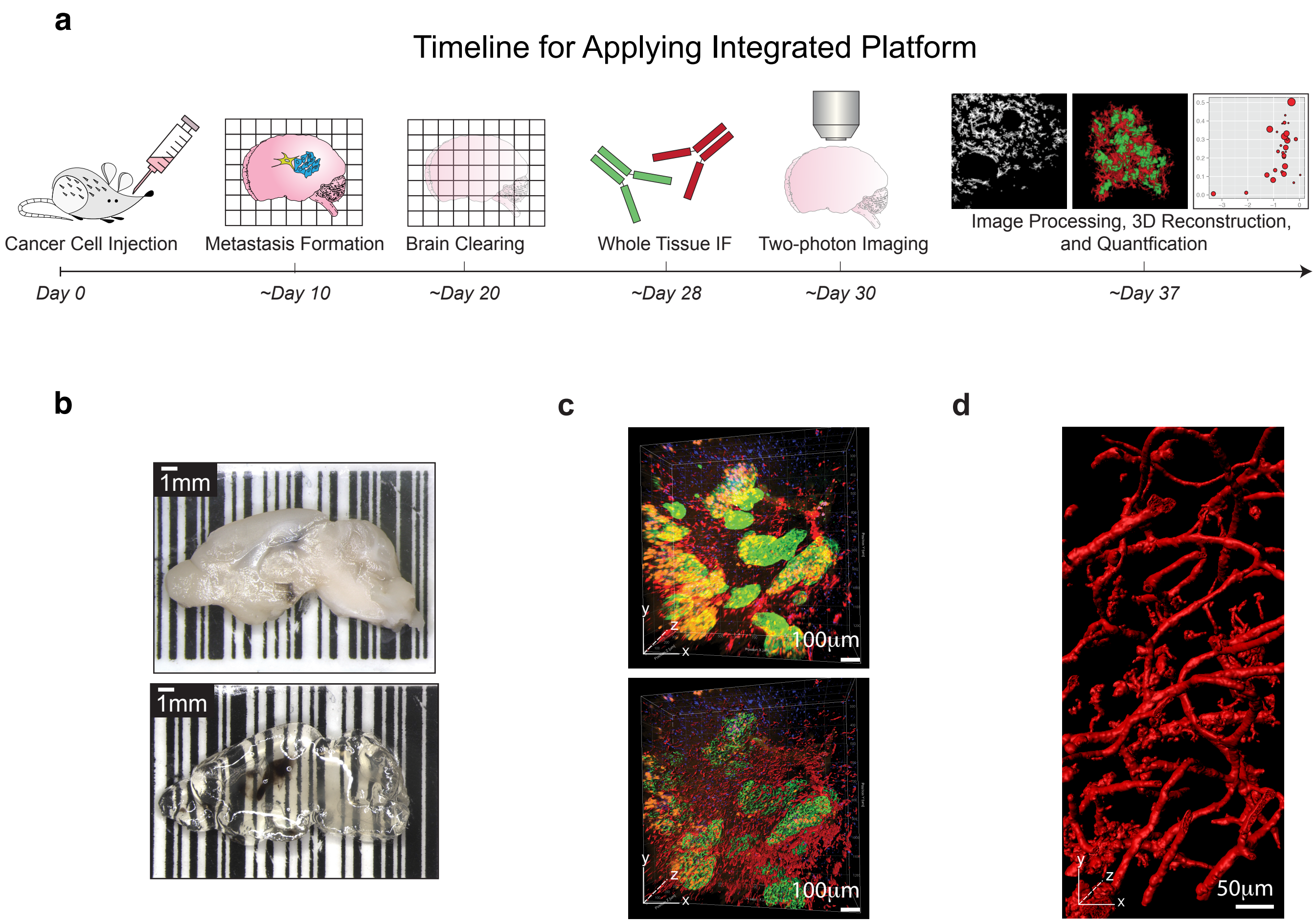

Supplementary Figure 2

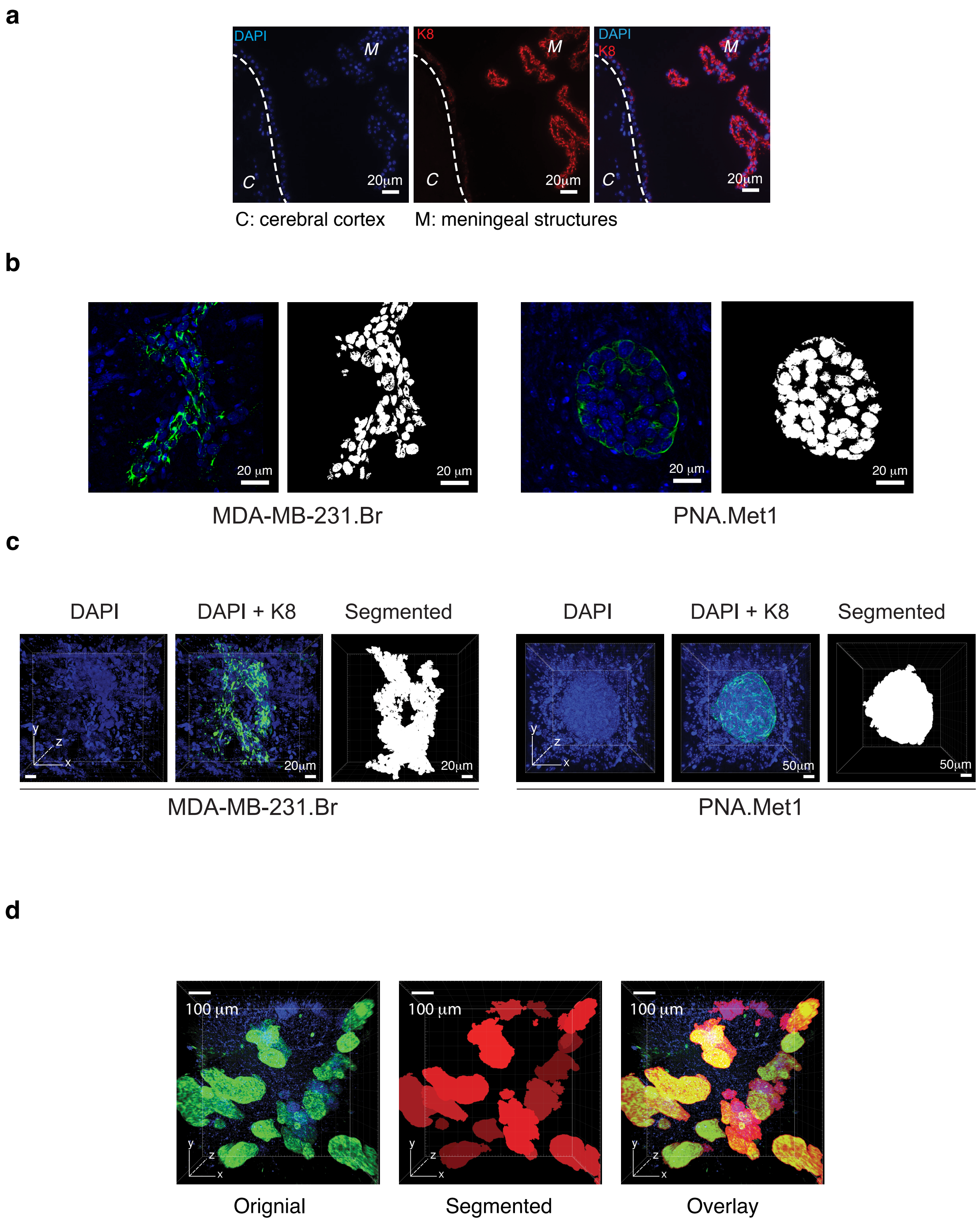

Supplementary Figure 3

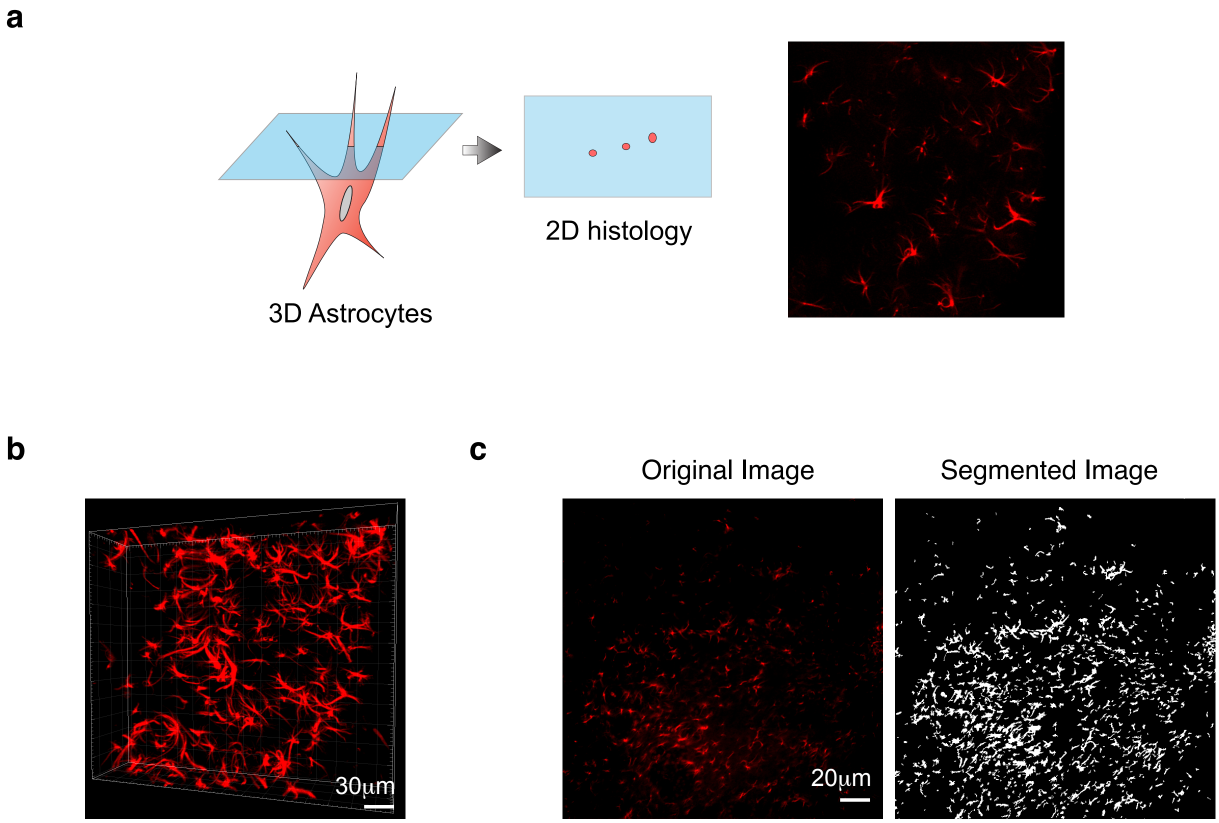

Supplementary Figure 4

a

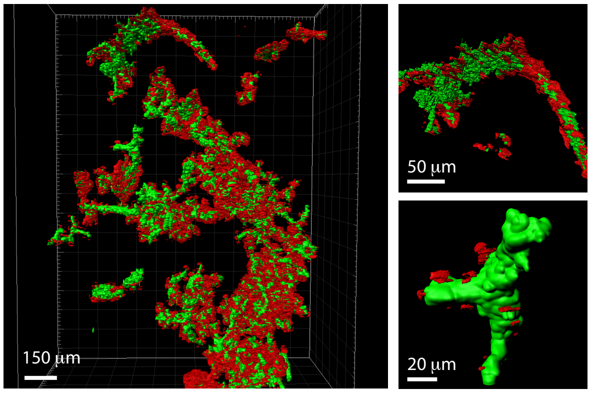

b

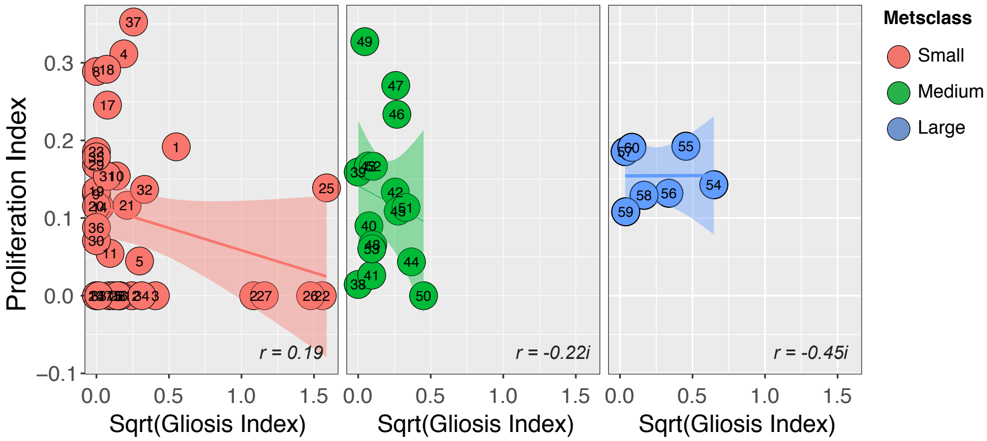

Supplementary Figure 5

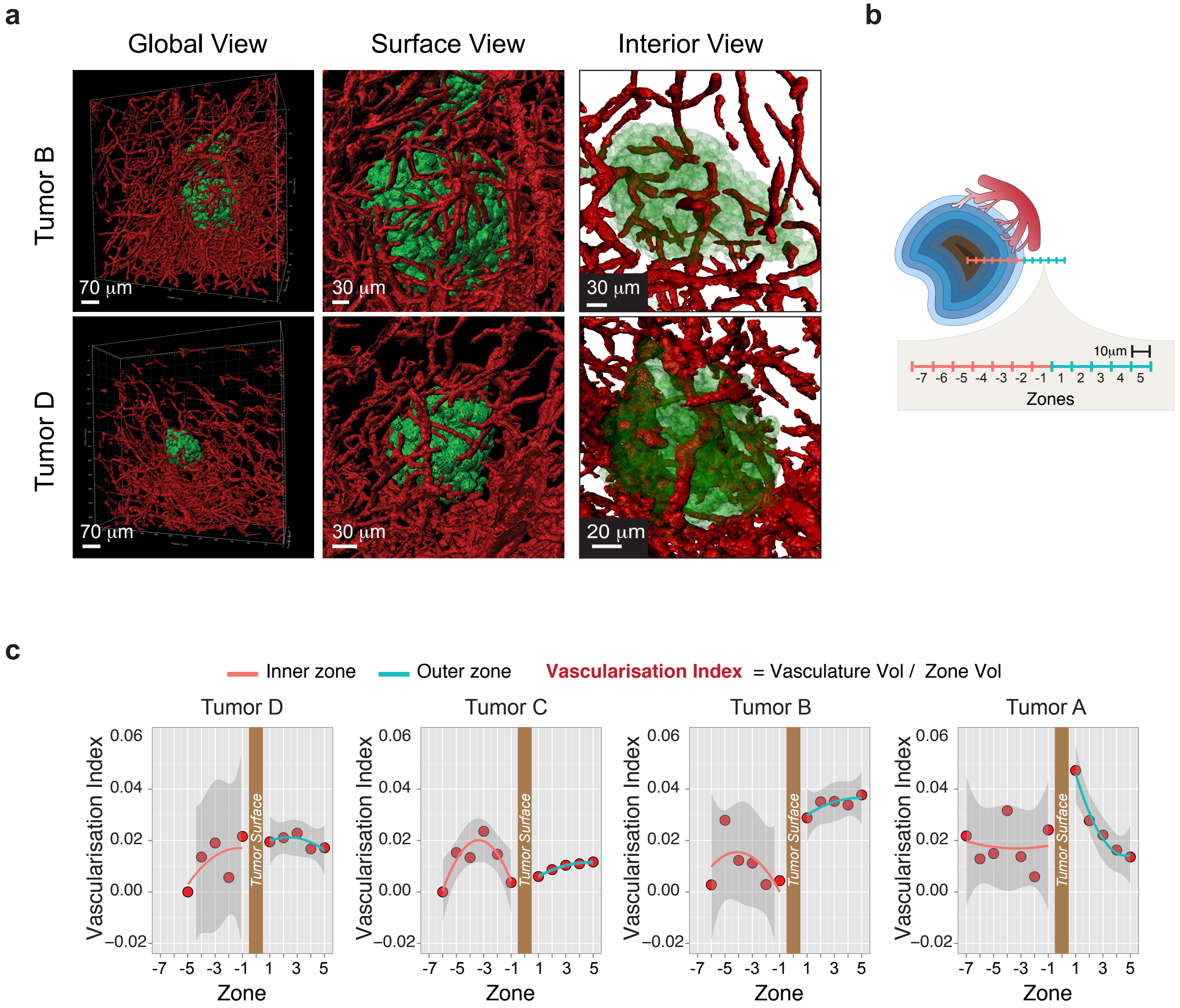

Supplement: Supplementary Information [file srep24201-s1.pdf]
